# Ultra-High-Performance Liquid Chromatography-High Resolution Mass Spectrometry and Gas Chromatography-Mass Spectrometry Screening of Classical Psychoactive Substances, Novel Psychoactive Substances, and Metabolites in Postmortem Urine Samples

**DOI:** 10.3390/molecules31193458

**Published:** 2026-09-28

**Authors:** Eleazar Vargas-Mena, Milton Rosero-Moreano, Gonzalo Taborda-Ocampo, Julio César España-Amortegui, Andrés F. González Suárez, Elena E. Stashenko

**Affiliations:** 1 Grupo de Investigación en Toxicología Forense y Ciencias Analíticas Relacionadas (CROMATOX), Laboratorio de Toxicología Forense, Instituto Nacional de Medicina Legal y Ciencias Forenses, Avenida Las Américas No 98-25, Pereira 660004, Colombia; 2 Grupo de Investigación en Cromatografía y Técnicas Afines (GICTA), Departamento de Química, Facultad de Ciencias Exactas y Naturales, Universidad de Caldas, Manizales 170004, Colombia; milton.rosero@ucaldas.edu.co (M.R.-M.); gtaborda@ucaldas.edu.co (G.T.-O.); 3 Research Group for Analytical Food Chemistry, Technical University of Denmark, 2800 Copenhagen, Denmark; jceam@food.dtu.dk; 4 Centro de Investigación en Biomoléculas (CIBIMOL), Escuela de Química, Universidad Industrial de Santander, Bucaramanga 680001, Colombia; andresgonzalez2285@gmail.com (A.F.G.S.); elenastashenko@gmail.com (E.E.S.)

**Keywords:** new psychoactive substances, mass spectrometry, urine screening, forensic drug screening, UHPLC-Orbitrap-HRMS, GC/MS, forensic toxicology

## Abstract

The use of new psychoactive substances (NPSs) is increasing, and their chemical diversity together with potential postmortem changes demands accurate screening and confirmatory analysis in biological matrices. This study highlights the analytical value of combining ultra-high-performance liquid chromatography-high-resolution mass spectrometry (UHPLC-ESI^(+/−)^-Orbitrap-HRMS) and gas chromatography-mass spectrometry (GC/MS) for the broad-coverage screening of classical psychoactive substances (CPSs), NPSs, and their metabolites in a selected cohort of 25 postmortem urine samples with suspected NPS consumption; given this limited size, results should be regarded as a proof-of-concept rather than epidemiologically representative. Forty-four drugs/metabolites were identified, most confirmed against authentic standards (Level 1), while a subset of minor or emerging metabolites received tentative/probable identifications (Level 2) based on accurate mass, isotopic pattern, and fragmentation. Ketamine, one of the most prevalent drugs (88% LC/MS, 84% GC/MS), followed by cocaine (68% LC/MS, 12% GC/MS); amphetamine-type substances (ATS) showed marked technique-dependent detection (e.g., 3,4-methylenedioxyamphetamine (MDA): 48% LC/MS, 8% GC/MS). LC/MS offered broader polarity coverage. GC/MS reliably detected parent drugs and derivatizable xenobiotics. Together, both platforms provided complementary confirmed and tentative-level coverage for forensic urine screening; quantitative validation and larger cohorts are needed before diagnostic, impairment, or epidemiological extrapolation can be made.

## 1. Introduction

The increase in the abuse of classical psychoactive substances (CPSs) and novel psychoactive substances (NPSs), especially anesthetics and synthetic opioids, has created a public health crisis. In several countries, the rates of deaths and intoxications related to this kind of drug use have risen, as well as phenomena related to criminality, adverse events, and mental health problems, which are particularly present in school-age teenagers and young adults [1,2,3].

Despite the opioid crisis in North America, it is essential to consider that there is not a unique NPS consumption global trend, and that these phenomena could shift according to social determinants, the type of drug, consumer, region, supply, and demand. There are distinct effects for different substances, each associated with specific compounds that trigger imbalances and dysregulation in pathways across several systems (e.g., dopaminergic, serotonergic, GABAergic). Each altered pathway could produce a distinctive pharmacological profile, which is why there is an imperative need for the forensic toxicology system to determine the best methodologies for the reliable identification of any kind of drugs and their metabolites [4,5,6,7], to help elucidate the mechanism of drug-impairment, intoxication, or death in the forensic toxicology system, and the generation of adverse event treatment in clinical toxicology and emergency medicine practice [8,9,10,11,12,13].

Several methods for extracting NPSs and their metabolites have been reported in the literature, including solid-phase extraction (SPE) using various sorbents. Traditional liquid extraction (LLE) and miniaturized, eco-friendly techniques such as hollow fiber solvent bar microextraction, liquid–liquid microextraction, membrane-protected molecularly imprinted polymers, and QuEChERS or dispersive extraction have also been reported [14,15,16,17,18,19]. Urine is the preferred matrix for forensic and clinical analysis due to its easy, non-invasive collection, a broader diagnostic window (compared to blood), relatively high concentrations of drugs and metabolites, and ease of extraction, often requiring hydrolysis and chemical derivatization before chromatographic analysis [20,21,22]. Regarding instrumental analysis, publications report the use of universal detectors for targeted screening, low mass-resolution GC and LC in single quadrupole in full-scan mode, as well as triple quadrupole systems (QqQ-MS) for targeted analysis and quantification, and high-resolution LC-MS/MS for both targeted and non-targeted analysis, focusing on the simultaneous confirmation and quantification of compounds using data-dependent and independent acquisition modes [23,24,25,26]. System selection must account for the physicochemical differences between the lipophilicity of Phase I metabolites and the hydrophilicity of Phase II metabolites; steps or modifications should be applied to improve extraction efficiency. Recently, several approaches have aimed to elucidate drug–drug interactions and potential metabolites using in silico ADMETox and quantitative structure–activity relationship models, as well as in vitro analyses using cell and animal models [27,28,29].

This study aimed primarily to implement two methodological setups for the screening of NPSs, CPSs, and their metabolites; the analytical performance of both GC/MS and UHPLC-ESI ^(+/−)^-Orbitrap-HRMS was evaluated according to ANSI/ASB 036-2019 Standard Practices for Method Validation in Forensic Toxicology for qualitative analysis [30]. The methods were applied to determine a selection of NPSs, CPSs, and their metabolites in urine using GC/MS and UHPLC-ESI ^(+/−)^-Orbitrap-HRMS as complementary analytical methods for screening in 25 urine samples provided by the Forensic Toxicology Laboratory at the National Institute of Legal Medicine and Forensic Sciences, with suspected consumption of ketamine, opiates, and ATS. Although both platforms have been independently applied to identify NPSs and CPSs in biological matrices and characterizations of tusibi, pink cocaine, or related slang names have been reported [31], to our knowledge, no study has specifically addressed the complementarity of these two technologies in forensic biological samples in our region. While GC/MS identified parent compounds, LC/MS provided a more comprehensive profile of urinary excretion products for both parent drugs and metabolites, including phase I and phase II metabolites, demonstrating the complementary value of the two techniques for rapid and reliable screening of CPSs and NPSs in urine. This qualitative approach helps expand the forensic metabolic chemical space and provides a starting point for linking forensic analytical toxicology with clinical and epidemiological practice by describing toxicological findings in relation to the recorded manner of death. These findings inform the need for future quantitative studies in blood to establish drug–drug interaction-related impairment and support harm-reduction strategies targeting the current consumption trends in the region.

## 2. Results

### Analysis of Forensic Samples by GC/MS and UHPLC-ESI ^(+/−)^-Orbitrap-HRMS

A dual platform screening approach was applied to urine samples from forensic casework in order to detect CPSs, NPSs, and metabolites. Compound identification met current forensic and analytical rigor, since detected features were categorized according to identification confidence levels adapted from Schymanski et al. and used by the Metabolomics Standards Initiative (MSI) [32,33]. Confirmed Structures (Level 1) were assigned when an authentic reference standard (listed in Table S1 with supplier lot numbers and exact chemical formulas) was analyzed within the same analytical sequence. Retention time, precursor accurate mass, and experimental MS/MS or EI mass spectral library matches were required for confirmation. Level 2 was assigned to molecules where commercial reference standards were unavailable (tentative identification). This category relied on the precursor mass accuracy along with experimental spectral library matching (similarity score) supported by the accuracy of two fragmentation products, which were individually tested. It is worth noting that isomeric metabolites are reported as Level 2 annotations that are not claimed as structural confirmation since accurate mass cannot distinguish positional or regio-isomeric patterns not resolved chromatographically. Selectivity assessment showed no coeluting interferents from endogenous or exogenous compounds at the retention time and with the same mass spectra as the drugs of interest. Carryover was negligible, as solvent, sample blanks, and negative controls showed no signals for CPSs, NPSs, or metabolites. Limits of detection ranged from 21 to 438 ng/mL and 8 to 41 ng/mL for GC/MS and LC/MS, respectively (see Table 1).

Table 2 details the xenobiotics of forensic interest identified in 25 screened samples and shows the informed cause of death. Identification data were anonymized to meet ethical research requirements.

Dissociative anesthetic ketamine was found in 22/25 (88%) and 21/25 (84%) of the samples by LC/MS and GC/MS, respectively. Norketamine, the main active metabolite, was detected in 19/25 (76%) and 5/25 (20%) of the samples by LC/MS and GC/MS, respectively. The hydroxylated forms, hydroxynorketamine and hydroxyketamine, were tentatively identified only by LC/MS in 14/25 (56%) and 16/25 (64%) of samples, respectively. Both are products of the hydroxylation of the cyclohexanone ring of ketamine and norketamine, respectively. Ketamine glucuronidation (phase II metabolism) was not detected under the present analytical conditions (Figure S1). Cocaine was identified in 17/25 (68%) and 4/25 (16%) of the samples for LC/MS and GC/MS, respectively. It is extensively biotransformed via both enzymatic and non-enzymatic pathways to the pharmacologically inactive benzoylecgonine (BE), detected in 23/25 (92%) and 13/25 (52%) of the samples by LC/MS and GC/MS, respectively, a product of cocaine hydrolysis catalyzed by carboxylesterases. Ecgonine methyl ester (EME), an inactive metabolite formed by enzymatic hydrolysis of cocaine, was detected in 9/25 (36%) and 8/25 (32%) of the samples by LC/MS and GC/MS, respectively. Ecgonine, an inactive metabolite produced by hydrolysis of BE and EME, was present in 9/25 (36%) of the samples analyzed by LC/MS. When cocaine and alcohol are used together, some cocaine undergoes transesterification, forming in the body at least two new substances. Cocaethylene was present in 8/25 (32%) of the samples for LC/MS. Ecgonine ethyl ester (EEE), an inactive metabolite formed during the hydrolysis of cocaethylene, was detected in 8/25 (32%) of the samples analyzed by LC/MS (see Figure S2) [34,35,36,37,38].

The hallucinogen Δ^9^-tetrahydrocannabinol, the main active constituent of Cannabis sativa, undergoes various phase I transformations until the formation of the main urinary inactive metabolite 11-nor-9-carboxy-Δ^9^-tetrahydrocannabinol THC-COOH, along with its phase II metabolite 11-nor-9-carboxy-Δ^9^-tetrahydrocannabinol-glucuronide, which were detected only by LC/MS in 14/25 (60%) and 1/25 (4%) of the samples, respectively [39,40].

Central nervous system stimulants, entactogens, and hallucinogens: MDMA was present in 7/25 (28%) and 3/25 (12%) of the samples for LC/MS and GC/MS, respectively. MDA was found in 12/25 (48%) and 2/25 (8%) samples by LC/MS and GC/MS, respectively. Both substances were also tentatively annotated indirectly through their hydroxylated forms: HHMA 8/25 (32%), HHA 4/25 (16%), HMA 3/25 (12%), and HMMA 2/25 (8%). Methamphetamine (MA) was identified in 2/25 (8%) of the LC/MS samples, and its main hepatic metabolites, 4-HA and 4-HMA, were detected in 5/25 (20%) and 1/25 (4%) of the samples analyzed by LC/MS. Figures S3 and S4 show the putative urinary metabolic products of ATS. Amphetamine was found in 3/25 (12%) of the samples by LC/MS [41,42].

Central nervous system depressants consisted mainly of the benzodiazepine oxazepam, an active metabolite of diazepam, nordazepam, and temazepam, which were present in 14/25 (56%) of the samples; midazolam and its metabolite α-hydroxymidazolam were found in 3/25 (12%) and 1/25 (4%) of the samples for LC/MS and GC/MS, respectively. Both the synthetic opioid fentanyl and the analog norfentanyl were present in 6/25 (24%) and 1/25 (4%) of the samples for LC/MS and GC/MS, respectively; the metabolites hydroxyfentanyl and hydroxynorfentanyl were found in one sample analyzed by LC/MS. Morphine-3-glucuronide, a phase II metabolite of morphine, was detected in 1 sample [43,44,45,46].

Two active substances known as adulterants, or cuttings, were detected. Lidocaine was found in 16/25 (64%) and 5/25 (20%) of the samples by LC/MS and GC/MS, respectively, and its main metabolite, hydroxylidocaine, in 7/25 (28%) samples analyzed by LC/MS. Caffeine was detected in 23/25 (92%) and 20/25 (80%) of the samples by LC/MS and GC/MS, respectively; its metabolite, dimethylxanthine, was identified by LC/MS analysis for 24/25 (96%) and 14/25 (56%) of the samples.

## 3. Discussion

Systematic toxicological analysis by full-scan GC/MS, combined with deconvolution and extensive low-resolution spectral libraries, has been the reference approach in forensic toxicology for decades. Its principal strengths—high reproducibility and broad library coverage, including silylated derivatives—are well documented [47,48]. Its principal limitations are equally well described: thermal lability of several analyte classes, the need to hydrolyze glucuronic adducts and derivatize polar metabolites, and limited sensitivity for highly polar and conjugated species [4,22]. LC/MS screening on Orbitrap and QTOF platforms was developed to address these constraints, and published urine screening methods report detecting several compounds using accurate-mass suspect lists, commercial, experimental, and in silico spectral libraries, and minimal sample preparation [49,50]. Our results are consistent with that body of work: the LC/MS platform returned a more complete urinary excretion profile, particularly for hydroxylated phase I metabolites and glucuronides, whereas GC/MS contributed orthogonal, EI-library-based evidence for parent compounds and for a subset of phase I metabolites. The methodological contribution of the present study is therefore not the introduction of a new platform, but the paired application of both platforms to the same authentic postmortem casework urine samples, which allows the discrepancy between the two techniques to be attributed to analyte chemistry rather than to differences in case populations, and the documentation of ketamine-centered polydrug patterns (“tusi”) in Colombian forensic casework.

Detection in urine is governed by the urinary detection window of each analyte and of its main metabolite, and not by any single elimination profile common to NPSs; reported half-lives among NPSs range from less than 1 h (e.g., cathinone) to more than 20 h for designer benzodiazepines (e.g., flubromazolam). For the substances detected here, the relevant considerations are compound-specific [51]. Ketamine has a relatively short half-life, whereas its N-demethylated metabolite, norketamine, and downstream hydroxylated species persist longer and are excreted in greater relative abundance, consistent with the higher detection frequency of norketamine, hydroxynorketamine, and hydroxyketamine than expected from the parent alone. Cocaine is rapidly hydrolyzed by carboxylesterases and plasma cholinesterase, so its detection in urine essentially marks very recent exposure, while benzoylecgonine and ecgonine methyl ester, with substantially longer half-lives, define the practical detection window; cocaethylene, formed only in the presence of ethanol, additionally documents concurrent alcohol use. ATS shows urinary-pH-dependent elimination, which limits inferences about dose or time of intake from detection frequency. THC-COOH, a terminal carboxylic metabolite excreted largely as its glucuronide, has by far the longest detection window and may reflect exposure days to weeks before death, particularly in long-term users. Consequently, differences between the time of ingestion and the time of sampling, together with analyte-specific ADME behavior and, in postmortem cases, the unknown survival interval, are expected to contribute to the observed pattern of parent-drug versus metabolite detection [52,53].

The *R*^2^ values obtained by GC/MS for several analytes—methamphetamine (0.478), nitrazepam (0.662), MDMA (0.732), thioridazine (0.792), clonazepam (0.794), MDA (0.839) and caffeine (0.841)—did not meet our predefined acceptance criterion, whereas the corresponding LC-HRMS values did. Rather than an intrinsic limitation of mass-spectrometric detection, this behavior reflects the cumulative yield of preceding steps, a pattern repeatedly described for GC/MS-based systematic toxicological analysis [54,55]. Cleavage of glucuronide and sulfate conjugates must precede extraction; enzymatic hydrolysis with β-glucuronidase is analyte-gentle but slow and incomplete for some conjugates, whereas acid or alkaline hydrolysis is faster but degrades labile analytes such as the 7-nitrobenzodiazepines and 6-monoacetylmorphine. Silylation of amino, hydroxy, and carboxylic functional groups is then required, and derivatization yields vary with steric hindrance, the electronic environment of the reactive site, water content, and reaction kinetics, so that a single set of conditions cannot be optimal across a panel spanning amphetamines, opioids, benzodiazepines, and cocaine metabolites [55]. Finally, thermally labile and adsorptive analytes may undergo partial degradation or rearrangement in the injection port and on active surfaces, producing non-reproducible mass transfer to the column and hence poor linearity [56,57,58]. In the present work, enzymatic hydrolysis and trimethylsilylation were selected as a compromise between simplicity, sensitivity, chemical coverage, reaction time, sample integrity, chromatographic behavior, and the availability of library spectra for TMS derivatives; the *R*^2^ values reported above delimit the analytes for which this compromise is unfavorable and for which LC/MS should be regarded as the method of preference.

Glucuronides were detected only by LC/MS, as expected. Conjugates carry additional hydroxyl and carboxyl functions that increase polarity and hydrogen-bonding capacity, which shortens retention on reversed-phase columns and, for acyl and ether glucuronides, reduces ionization efficiency under standard positive-mode ESI conditions; negative-mode acquisition and dedicated chromatographic conditions are commonly recommended for their determination [59]. In GC/MS, conjugated analytes are not observed as such by design: they contribute to the signal of the deconjugated parent or phase I metabolite after hydrolysis, so direct evidence of conjugation is inaccessible on that platform. This distinction is analytically relevant because direct detection of the intact conjugate—as obtained here for 11-nor-9-carboxy-Δ^9^-THC-glucuronide, nordazepam-glucuronide, and morphine-3-glucuronide—provides evidence of metabolic transformation in vivo that survives independently of hydrolysis efficiency, an argument advanced in favor of hydrolysis-free LC/MS screening [60]. The transformation products annotated here are consistent with the sequential oxidation, hydroxylation, and conjugation pathways described for these compound classes [61,62].

Four observations have practical consequences for forensic laboratories in the region. First, the preliminary polydrug pattern documented in Table 2 (e.g., ketamine–ATS-cocaine–caffeine), and, in particular, the frequency with which ketamine and its metabolites appear in casework locally described as “tusi”, indicates that screening panels that do not include ketamine metabolites will potentially misclassify these cases [63]. Second, the detection of lidocaine and caffeine alongside cocaine, ketamine, and its metabolites is consistent with the adulteration profiles reported for samples seized or reported worldwide [31]; for caffeine, however, the analytical finding is not interpretable in isolation, because dietary intake is ubiquitous in the Colombian population and caffeine and its metabolites, theobromine and 1,3,7-trimethyluric acid, were present in nearly all samples. Caffeine should therefore be reported as a finding of pharmacological rather than forensic significance, relevant mainly to drug–drug interactions, unless supported by the seized-material analysis of the corresponding case. Third, screening identifies substances, prior exposure, and urinary excretion hypotheses, but does not establish their contribution to death; once a xenobiotic or metabolite is identified, quantification in blood using certified reference materials and a fully validated quantitative method remains indispensable for assessing impairment and toxic or lethal exposure. Given the speed with which the NPS market changes, spectral libraries, accurate-mass suspect lists, and screening panels require continuous updating [64,65,66]. Fourth, despite the enrichment of this cohort toward ketamine/ATS positive casework, no NPSs in the strict sense were confirmed or tentatively identified: no synthetic cathinones, no benzimidazole/nitazene opioids, no designer benzodiazepines, and no phenethylamines such as 2C-B, despite the latter being repeatedly reported as a component of “tusi”-branded mixtures in the region [31]. This no-detection finding should not be read as evidence of their absence from the local drug supply; the ever-changing nature of these drugs creates the need to expand the analytical portfolio of the forensic laboratory.

The present study used a purposive sampling strategy: only casework screened presumptive positive for NPSs, CPSs, or metabolites by immunoassay were included. This enrichment was deliberate and appropriate for the study objective but introduces an unavoidable verification bias. Consequently, data reported here should not be interpreted as prevalence. Further research should address the need for quantitative analysis, using a larger sample size for prevalence and causality interpretation, and include a broad panel of biological matrices (blood, bile, vitreous humor) to assess matrix effects in recoveries.

## 4. Materials and Methods

### 4.1. Reagents and Chemicals

Acetonitrile, methanol, and ethanol of analytical grade were purchased from Mallinckrodt (Dublin, Ireland); dichloromethane was purchased from Merck (Darmstadt, Germany), and ultrapure helium 99.995% (Messer—Bucaramanga, Colombia) was used as the mobile phase in chromatographic analysis.

All standard solutions for quality control were prepared at a concentration of 10 mg mL^−1^ (stock solution) and then diluted to a working solution of 1 mg mL^−1^. Table S1 lists potential target drugs or metabolites to monitor during analysis, including their molecular formula, retention time (*t_R_*), and experimental exact and average masses.

### 4.2. Sample Preparation by GC/MS and UHPLC-ESI ^(+/−)^-Orbitrap-HRMS

Figure 1 reports the workflow for urine analysis in both methodologies. A total of 80 authentic human urine samples of forensic origin were provided by the National Institute of Legal Medicine and Forensic Sciences, Regional Office of Pereira, corresponding to casework received during forensic procedures. Sample selection followed a purposive design intended to maximize the number of samples containing the target analytes, and was therefore not designed to estimate the prevalence of these substances in the forensic population. All samples were fully anonymized and internally coded before selection; the analyst had no access to personal identifiers, case data, or demographic information. Urine samples were previously qualitatively screened (presence/absence) by homogeneous enzyme immunoassay (EMIT^®^, Xerion, Bogotá, Colombia) for drugs of abuse and their metabolites, namely cocaine (as benzoylecgonine), opiates, cannabinoids (as THC-COOH), benzodiazepines, ATS (as MDMA or amphetamine), methadone, ketamine, and barbiturates. Exclusion criteria included samples with a medicolegal autopsy performed more than 48 h after death, negative (absence) EMIT results, samples with volumes below 10 mL, and samples showing visible degradation or adulteration. A total of 25 urine samples tested preliminary positive for ketamine or ATS. Aliquots of 6 mL were taken and stored at −20 °C until analysis. Two extraction methodologies were applied. Extraction methods were based on the forensic laboratory’s protocols, slightly modified to enable the identification of new molecules and their metabolites [67,68,69]. For confirmatory GC/MS analysis, 100 μL of flurazepam (ISTD) was added to 2 mL of the forensic sample, followed by 300 μL of sodium acetate buffer pH 4.5, then 50 μL of β-glucuronidase (*Helix pomatia*), and incubation at 56 °C for 2.5 h. The pH was adjusted to 10 by adding 500 μL of sodium tetraborate buffer, followed by 6 mL of dichloromethane. The mixture was vortexed for 1 min, then centrifuged at 2490× *g* for 10 min. The organic phase was dried under a gentle nitrogen flow, and 50 μL of BSTFA+TMCS mixture (99:1) and 50 μL ethyl acetate were added to the dry extract, followed by incubation at 80 °C for 20 min. A total of 1 μL was injected into the GC/MS in full scan mode. For UHPLC-ESI ^(+/−)^-Orbitrap-HRMS analysis, 20 μL of oxazepam-d5 (ISTD) was added to 200 μL of urine, followed by 600 μL of ice-cold acetonitrile (ACN), vortex agitation for 1 min, and centrifugation for 10 min at 2490× *g*. The supernatant was dried under a gentle stream of nitrogen, then reconstituted with mobile phase, and 2 μL was injected into the liquid chromatograph.

To check the reliability of the two methodologies, sample runs included: solvent blank, used between sample injection to clean the system and avoid carryover; matrix blank, to determine the presence of endogenous compounds that may co-elute with the analytes of interest, and isobars that could produce false positive; negative control in a real matrix, to discard solvent, reactants and system contamination, assess drifts in retention time (due to internal standard), and a positive control in both aqueous and real matrix samples enriched with reference materials at final concentrations of 100 ng/mL for LC/MS and 1000 ng/mL for GC/MS, to estimate shifts in retention times, the efficiency of the extraction, the detection capacity of the libraries and establish a contrast medium. Pooled Quality Control Samples (Pool QCs) were prepared by mixing 20 µL aliquots of each sample to assess the repeatability of retention times and analytical response. Controls were treated as samples and analyzed in the same analytical sequence as the study samples, using descriptive univariate statistics to characterize the method’s analytical performance (see Section 4.5). No inferential statistical tests were applied because the objective was to characterize analytical precision rather than test differences between independent samples.

### 4.3. Gas Chromatography–Mass Spectrometry Analysis

GC/MS analysis was conducted on a gas chromatograph (GC 7890A System Plus, Agilent Technologies, Palo Alto, CA, USA) coupled with a selective mass detector (Agilent Technologies, MSD 5975C) using electron ionization at 70 eV. NPSs and their metabolites were separated on a capillary column with 5–phenyl-95% poly (dimethylsiloxane) (DB-5MS 30 m × 250 μm I.D., 0.25 μm film thickness, J&W Scientific, Folsom, CA, USA). The ionization source and interface temperatures were 230 °C and 300 °C, respectively. Injection was performed via pulsed splitless mode at 270 °C in the injection port. Full-scan data were acquired over a mass range of 40–450 *m*/*z* at a rate of 3.58 scans per second. The oven was initially set to 80 °C for 1 min, then heated at 15 °C min^−1^ to 200 °C for 5.3 min, followed by heating at 10 °C min^−1^ to 300 °C for 12 min. The total run time was 38.3 min. Standard solutions of the analytes were prepared in an authentic matrix at 0.5 μg/mL. Data analysis involved an automated workflow using Agilent MSD ChemStation Software (Ver. F.01.03.2357, Agilent Technologies, Santa Clara, CA, USA) for mass spectral deconvolution, peak detection, base peak integration, and compound identification via comparison with the NIST 2023 library and an in-house library containing *t_R_* and *m*/*z* of confirmed reference standards.

### 4.4. Ultra-High-Performance Liquid Chromatography-High-Resolution Mass Spectrometry Analysis

The urine extracts were analyzed in a VanquishTM ultra-high-performance liquid chromatograph (UHPLC, Thermo Scientific, Waltham, MA, USA), equipped with a degassing unit, a gradient binary pump, and an autosampler maintained at 10 °C. A Zorbax Eclipse XDB C18 chromatographic column (Sigma Aldrich, St. Louis, MO, USA) of 50 mm length × 2.1 mm I.D. and 1.8 μm particle diameter was used. The flow rate of the mobile phases containing Type I water (A) and methanol (B), both containing ammonium formate (5 mM) and formic acid (0.1%), was 300 μL min^−1^. The elution gradient program was as follows: 100% A for 8 min, then 100% B for 5 min, followed by 100% A for 7 min. The total run was 20 min, and the injection volume was 2 μL. The UHPLC was connected to a Q-Exactive Plus Orbitrap mass spectrometer (Thermo Scientific, Bremen, Germany) with a heated electrospray ionization source (HESI-II) and polarity switching for periods <500 ms, with HCD fragmentation. The capillary voltage was 3.5 kV. The nebulizer and capillary temperatures were 350 °C and 320 °C, respectively. The mass resolution was set to 70,000 (full width at half maximum, FWHM) at *m*/*z* 200, with an automatic gain control target of 3 × 106, a maximum C-trap injection time of 200 ms, and a mass range of *m*/*z* 80–1000. The ions injected into the HCD via the C-trap were fragmented with normalized collision energies from 10 eV to 40 eV. Mass spectra were collected in all-ion fragmentation mode for each collision energy, employing a mass resolution of 35,000. The instrument was fully calibrated using the Pierce LTQ Velos ESI positive ion calibration solution and the Pierce ESI negative ion calibration solution (Thermo Scientific, Rockford, IL, USA). Data were analyzed using Thermo Xcalibur 3.1 software (Ver. 4.6.67.17, Thermo Scientific, San Jose, CA, USA) and Compound Discoverer (Ver. 3.4, Thermo Scientific). The compounds were identified by comparing the *t_R_* and accurate mass (Δppm < 1) with standard substances prepared in an authentic matrix at 0.1 μg mL^−1^, analyzed in the same batch as the samples, and those reported in the local compound database and NIST 2020 library. Additional putative metabolite identifications were performed by comparing the observed exact masses of each molecule with those reported in NIST 2023 and in silico platforms, including mzCloud, CFM-ID, ChemSpider, DrugBank, and MassBank of North America [67,68,69,70].

### 4.5. Analytical Performance

Method performance for both methodologies was evaluated according to ANSI/ASB 036-2019 Standard Practices for Method Validation in Forensic Toxicology for qualitative analysis. Linear calibration curves (*n* = 3) were calculated using an ordinary least squares (OLS) model. Blank urine aliquots were spiked with the stock solution to obtain calibration samples in real matrix at nominal concentrations of 1, 15, 30, 60, 125, 250, 500, and 1000 ng/mL for LC/MS and 15, 30, 60, 125, 250, 500, and 1000 ng/mL for GC/MS. Replicates (*n* = 3) at each concentration were analyzed as described above. After visually inspecting the signal-concentration plot, we statistically evaluated, for 3 independent calibration curves, the coefficient of determination (*R*^2^), mean absolute error, slope of regression (*b*), and intercept (*a*). Calibrations were accepted after evaluation of the statistical significance for the assumptions of *R*^2^ ≥ 0.9, *b* ≠ 0, and *a* = 0. LODs for LC/MS were calculated statistically from the following equation:

(1) $$LOD=3.3\times \frac{s}{b}$$

where *s* is the residual standard deviation of the intercept Sa, and *b* is the calibration graph slope. Complementary, one working solution at a concentration of the LOD was prepared and used as part of the run’s quality control for each methodology. Table 1 shows the analytical performance results for all the CPSs, NPSs, and metabolites.

Carryover was addressed continuously as part of the run’s quality assurance by injecting solvent blanks after every sample, especially positive controls. Solvent blanks were required to show no detectable signal at the characteristic retention time of the target analytes. Selectivity was assessed against matrix blanks, monitoring the absence of interferers that affect identification. Negative controls in authentic matrix were used to monitor the absence of false positives throughout the assessment of characteristic ions at the *t_R_* of the target analytes. Positive controls were used to retrieve the expected mass spectra and confirm identification at the corresponding *t_R_* for each target compound. Pooled QC samples were used to assess analytical performance. For both pooled QC and positive controls, the mean, standard deviation, and relative standard deviation (%RSD) of Retention time, mass accuracy, and mass-error drift were monitored for injections across the analytical sequence (acceptance criterion: *t_R_* drift ± 0.1 min, base peak ion present, sample area and *t_R_* ≤ 20% RSD, Δppm < 1 (LC/MS). Figures S5 and S6 show a visual representation of quality assurance for the analytical run, assessing repeatability in normalized base-peak areas of ketamine for GC/MS and LC/MS.

## 5. Conclusions

The present study demonstrates the feasibility of a complementary UHPLC-ESI^(+/−)^-Orbitrap-HRMS and GC/MS strategy for the qualitative detection of selected CPSs, NPSs, and metabolites in urine. The methods were successfully applied to 25 authentic postmortem urine samples, providing useful information on the presence of multiple substances in the investigated samples and potentially contributing to preliminary toxicological characterization in postmortem investigations. The co-detection of multiple drugs or metabolites in the urine should, however, be interpreted primarily as evidence consistent with exposure and urinary excretion; this study does not establish drug–drug interactions, impairment, or a causality between drug exposure and death. Such interpretations require quantitative blood analysis, along with relevant background and forensic evidence.

Overall, the findings demonstrate the potential applicability of this approach as a complementary screening strategy in forensic toxicology. Further studies involving a broad group of molecules (e.g., synthetic cathinones, nitazene opioid) potentially present in the region, quantitative measurements in blood samples are needed to establish its analytical performance and applicability across different forensic and clinical contexts.

## Figures and Tables

**Figure 1:**
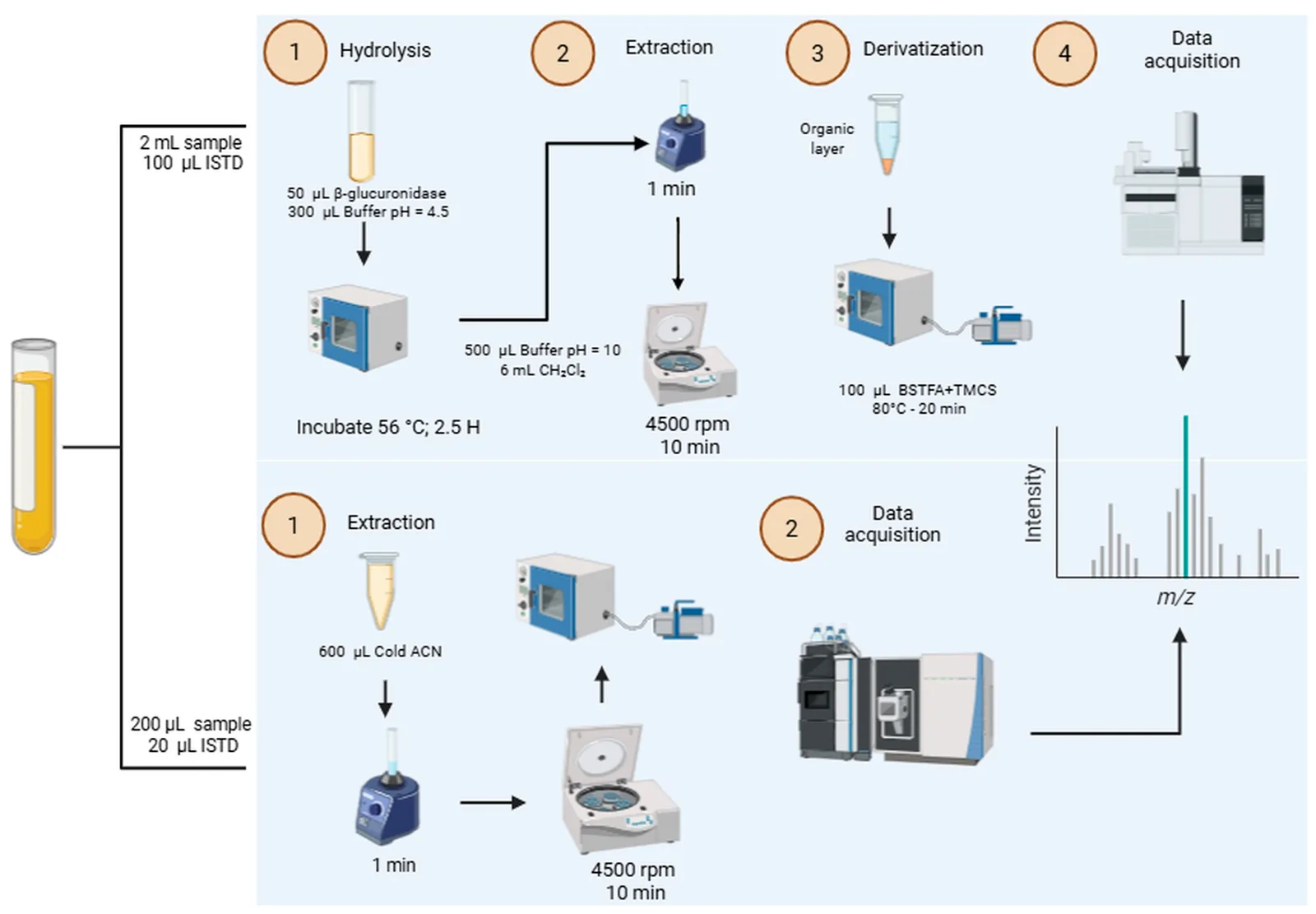
Workflow for urine screening analysis. Oxazepam-d5 was used as an internal standard for LC/MS, and flurazepam for GC/MS.

**Table 1:** Analytical performance results for CPSs, NPSs, and metabolites.

Compound	GC/MS		UHPLC-ESI ^(+/−)^-Orbitrap-HRMS	
	*R* ^2^	LOD ng/mL	*R* ^2^	LOD ng/mL
MDA	0.839	183	0.985	28
MDMA	0.732	253	0.983	30
6-Monoacetylmorphine	0.954	114	0.991	16
7-aminoclonazepam	0.931	143	0.985	31
7-aminoflunitrazepam	0.961	105	0.991	17
Alprazolam	0.921	153	0.986	29
Amitriptyline	0.985	62	0.987	27
Amphetamine	0.962	83	0.986	30
Benzocaine	0.953	116	0.987	29
Benzoylecgonine	0.963	103	0.992	8
Bromazepam	0.908	166	0.989	35
Caffeine	0.841	227	0.908	41
Carbamazepine	0.914	160	0.987	19
Chlorpromazine	0.949	121	0.991	25
Clonazepam	0.794	266	0.968	35
Clozapine	0.947	123	0.991	24
Cocaethylene	0.931	143	0.981	18
Cocaine	0.988	46.26	0.990	10
Codeine	0.996	25	0.992	22
Diazepam	0.983	68	0.982	26
Diltiazem	0.972	89	0.992	13
Dimethyltryptamine	0.918	156	0.997	18
Ecgonine	0.824	242	0.941	35
Ecgonine methyl ester	0.925	148	0.985	17
Ethylmorphine	0.997	21	0.991	15
Fentanyl	0.986	48	0.991	23
Hydrocodone	0.974	67	0.993	12
Hydromorphone	0.955	90	0.991	24
Imipramine	0.930	143	0.952	23
Ketamine	0.977	78	0.987	10
Levomepromazine	0.987	59	0.995	20
Lidocaine	0.983	69	0.994	9
Lorazepam	0.947	123	0.981	27
Meperidine	0.996	24	0.991	11
Methadone	0.990	41	0.991	25
Methamphetamine	0.478	438	0.986	28
Midazolam	0.946	124	0.991	17
Morphine	0.994	32	0.991	14
Nitrazepam	0.662	373	0.975	38
Nordazepam	0.930	143	0.967	24
Norketamine	0.967	96.	0.991	17
Oxazepam	0.939	132	0.984	17
Oxycodone	0.968	76	0.991	13
Papaverine	0.963	81	0.987	13
Phencyclidine	0.983	67	0.996	19
Temazepam	0.974	85	0.987	14
Thioridazine	0.792	269	0.987	16
Tramadol	0.981	59	0.997	10
Triazolam	0.923	151	0.985	17
Zolpidem	0.896	178	0.993	13

**Table 2:** Detailed GC/MS and UHPLC-ESI ^(+/−)^-Orbitrap-HRMS urine screening findings.

Code	Manner of Death	GC/MS ^a^	LC/MS
04SUP23	Suicide	BE-2TMS, caffeine, cocaine, EME-TMS	4-HA ^c^, caffeine ^b^, dimethylxanthine ^c,^ HNK ^c^, HK ^c^, ketamine ^b^, NK ^b^
665PEI23	Homicide	caffeine, ketamine, NK	BE ^b^, caffeine ^b^, cocaine ^b^, dimethylxanthine ^c^, EME ^b^, HNK ^c^, HK ^c^, HMA ^c^, HMMA ^c^, ketamine ^b^, MDMA ^b^, NK ^b^, oxazepam ^b^, THC-COOH ^b^
03PEI23	Homicide	caffeine, BE-2TMS, cocaethylene, EME-TMS, ketamine, MDA, NK	4-HA ^c^, 4-HMA ^c^, BE ^b^, caffeine ^b^, cocaine ^b^, dimethylxanthine ^c^, HNK ^c^, HK ^c^, ketamine ^b^, lidocaine ^b^, MA ^b^, NK ^b^, oxazepam ^b^, THC-COOH ^b^
187MZ23	Suicide	BE-2TMS, caffeine, cocaine, EME-TMS, ketamine, MDMA	amphetamine ^b^, BE ^b^, caffeine ^b^, cocaethylene ^b^, dimethylxanthine ^c^, ecgonine ^b^, EEE ^b^, EME ^b^, HMA ^c^, HMMA ^c^, HHMA ^c^, MDA^b^
265AR23	Homicide	ketamine	BE ^b^, cocaethylene ^b^, ketamine ^b^, NK ^b^
142MZ23	Suicide	caffeine, ketamine	BE ^b^, caffeine ^b^, cocaine ^b^, dimethylxanthine ^c^, HK ^c^, ketamine ^b^, NK ^b^, oxazepam ^b^
705PEI22	Traffic accident	α-hydroxymidazolam, caffeine, EME-TMS, fentanyl, ketamine, lidocaine, midazolam	α-hydroxymidazolam ^b^, BE ^b^, caffeine ^b^, cocaine ^b^, cocaethylene ^b^, dimethylxanthine ^c^, ecgonine ^b^, EME ^b^, fentanyl ^b^, HNK ^c^, HK ^c^, hydroxyfentanyl ^c^, hydroxynorfentanyl ^c^, hydroxylidocaine ^c^, ketamine ^b^, lidocaine ^b^, midazolam ^b^, nordazepam-glucuronide ^c^, NK ^b^, norfentanyl ^b^, oxazepam ^b^, THC-COOH ^b^
04PEI23	Suicide	BE-2TMS, caffeine, EME-TMS, ketamine, lidocaine, NK	α-hydroxymidazolam ^b^, BE ^b^, caffeine ^b^, cocaine ^b^, cocaethylene ^b^, dimethylxanthine ^c^, ecgonine ^b^, EEE ^b^, EME ^b^, HNK ^c^, HK ^c^, hydroxylidocaine ^c^, ketamine ^b^, lidocaine ^b^, midazolam ^b^, nordazepam-glucuronide ^c^, NK ^b^, norfentanyl ^b^, oxazepam ^b^, THC-COOH ^b^
757PEI22	Homicide	BE-2TMS, caffeine, ketamine, lidocaine	BE ^b^, caffeine ^b^, dimethylxanthine ^c^, HNK ^c^, HK ^c^, ketamine ^b^, MDA ^b^, NK ^b^, oxazepam ^b^, THC-COOH ^b^, THC-glu ^c^
244PEI23	Homicide	caffeine, ketamine	BE ^b^, cocaine ^b^, caffeine ^b^, dimethylxanthine ^c^, HK ^c^, HNK ^c^, ketamine ^b^, nordazepam-glucuronide ^c^, NK ^b^, oxazepam ^b^
129ARM23	Homicide	caffeine, ketamine	BE ^b^, caffeine ^b^, dimethylxanthine ^c^, ecgonine ^b^, hydroxylidocaine ^c^, ketamine ^b^, lidocaine ^b^, NK ^b^, oxazepam ^b^, THC-COOH ^b^
020SUP23	Homicide	BE-2TMS, caffeine, cocaine, cocaethylene, EME-TMS, ketamine, MDMA	BE ^b^, caffeine ^b^, cocaethylene ^b^, cocaine ^b^, dimethylxanthine ^c^, ecgonine ^b^, EEE ^b^, EME ^b^, HNK ^c^, HK ^c^, HHMA ^c^, hydroxylidocaine ^c^, ketamine ^b^, lidocaine ^b^, MA ^b^, MDA ^b^, NK ^b^, oxazepam ^b^, THC-COOH ^b^
697PEI23	Homicide	BE-2TMS, caffeine	BE ^b^, caffeine ^b^, dimethylxanthine ^c^, cocaethylene ^b^, cocaine ^b^, EEE ^b^, EME ^b^, HHMA ^c^, THC-COOH ^b^
045SUP22	Traffic accident	caffeine, ketamine	caffeine ^b^, dimethylxanthine ^c^, BE ^b^, cocaine ^b^, hydroxylidocaine ^c^, HHMA ^c^, lidocaine ^b^, oxazepam ^b^, THC-COOH ^b^
185PEI22	Undetermined	BE-2TMS, caffeine, cocaine, EME-TMS, hydromorphone, ketamine, methaqualone, morphine-2TMS	4-HA ^c^, BE ^b^, caffeine ^b^, cocaine ^b^, codeine ^b^, dimethylxanthine ^c^, HHMA ^c^, HNK ^c^, HK ^c^, hydromorphone ^c^, ketamine ^b^, methaqualone ^c^, morphine ^b^, morphine-3-glucuronide ^c^, NK ^b^, norhydromorphone ^c^, oxazepam ^b^
709PEI23	Traffic accident	caffeine, ketamine, lidocaine, NK	caffeine ^b^, cocaine ^b^, dimethylxanthine ^c^, fentanyl ^b^, HHMA ^c^, HK ^c^, HNK ^c^, hydroxylidocaine ^c^, ketamine ^b^, NK ^b^, lidocaine ^b^, MDA ^b^, nordazepam-glucuronide ^c^, norfentanyl ^b^, oxazepam ^b^, THC-COOH ^b^
370PEI23	Homicide	BE-2TMS, caffeine, EME-TMS, ketamine, lidocaine	BE ^b^, caffeine ^b^, cocaine ^b^, dimethylxanthine ^c^, fentanyl ^b^, HNK ^c^, HK ^c^, HMA ^c^, ketamine ^b^, levetiracetam ^c^, lidocaine ^b^, MDA ^b^, MDMA ^b^, NK ^b^, norfentanyl ^b^, THC-COOH ^b^
03CTG23	Homicide	BE-2TMS, caffeine	BE ^b^, caffeine ^b^, cocaine ^b^, cocaethylene ^b^, dimethylxanthine ^c^, ecgonine ^b^, EEE ^b^, EME ^b^, HHMA ^c^, HNK ^c^, HK ^c^, hydroxylidocaine ^c^, hydroxynorfentanyl ^c^, ketamine ^b^, lidocaine ^b^, MDA ^b^, NK ^b^, norfentanyl ^b^, oxazepam ^b^, THC-COOH ^b^
666PEI23	Traffic accident	caffeine	BE ^b^, caffeine ^b^, cocaine ^b^, dimethylxanthine ^c^, hydroxylidocaine ^c^, HHA ^c^, ketamine ^b^, lidocaine ^b^, nordazepam-glucuronide ^c^, norfentanyl ^b^, oxazepam ^b^, THC-COOH ^b^
153PEI23	Homicide	caffeine, codeine-TMS, ketamine, MDA, MDMA, NK	amphetamine ^b^, BE ^b^, cocaine ^b^, cocaethylene ^b^, dimethylxanthine ^c^, EME ^b^, HK ^c^, HNK ^c^, ketamine ^b^, lidocaine ^b^, MDA ^b^, MDMA ^b^, NK ^b^, THC-COOH ^b^
275PEI23	Suicide	ketamine	BE ^b^, caffeine ^b^, cocaine ^b^, dimethylxanthine ^c^, ketamine ^b^, lidocaine ^b^, MDA ^b^, MDMA ^b^, midazolam ^b^, oxymorphone ^c^
014PEI24	Homicide	BE-2TMS, caffeine, ketamine	4-HA ^c^, HHA ^c^, BE ^b^, caffeine ^b^, dimethylxanthine ^c^, ecgonine ^b^, EEE ^b^, EME ^b^, ketamine ^b^, lidocaine ^b^, MDA ^b^, NK ^b^, oxymorphone ^c^
379ARM23	Undetermined	Ketamine	4-HA ^c^, amphetamine ^b^, BE ^b^, caffeine ^b^, dimethylxanthine ^c^, ecgonine ^b^, EEE ^b^, ketamine ^b^, lidocaine ^b^, MDA ^b^, MDMA ^b^, midazolam ^b^, oxymorphone ^c^
102DOR23	Homicide	BE-2TMS, ketamine	BE ^b^, caffeine ^b^, cocaine ^b^, dimethylxanthine ^c^, ecgonine ^b^, EEE ^b^, HHA ^c^, HHMA ^c^, HK ^c^, HNK ^c^, ketamine ^b^, lidocaine ^b^, MDA ^b^, MDMA ^b^, NK ^b^, oxycodone ^c^, oxymorphone ^c^
396ARM23	Homicide	BE-2TMS, ketamine	BE ^b^, caffeine ^b^, dimethylxanthine ^c^, HHA ^c^, HK ^c^, HNK ^c^, ketamine ^b^, lidocaine ^b^, midazolam ^b^, MDA ^b^, MDMA ^b^, NK ^b^

^a^ All GC/MS identifications, except methaqualone and hydromorphone, correspond to level 1. ^b^ Confirmatory identification (level 1). ^c^ Tentative identification (level 2). 11-nor-9-carboxy-Δ^9^-tetrahydrocannabinol THC-COOH, 11-Hydroxy-Δ^9^-THC-glucuronide THC-glu, 4-hydroxyamphetamine 4-HA, 4-hydroxymethamphetamine 4-HMA, 3,4-dihydroxyamphetamine HHA, 3,4-methylenedioxymethamphetamine MDMA, 3,4-methylenedioxyamphetamine MDA, 4-hydroxy-3-methoxyamphetamine HMA, 4-hydroxy-3-methoxymethamphetamine HMMA, 3,4-dihydroxymethamphetamine HHMA, benzoylecgonine BE, ecgonine ethyl ester EEE, ecgonine methyl ester EME, Hydroxyketamine HK, hydroxynorketamine HNK, Norketamine NK, methamphetamine MA, trimethylsilyl adduct-TMS.

## Data Availability

All data generated or analyzed during this study are included in this published article.

## Supplementary Materials

The following supporting information can be downloaded at: https://www.mdpi.com/article/10.3390/molecules31193458/s1, Table S1: List of potential drugs or metabolites to monitor. Retention times (*t_R_*) and monitored ions (*m*/*z*) used for the screening gas chromatography-mass spectrometry (GC/MS) and ultra-high-performance liquid chromatography-high-resolution mass spectrometry analysis of forensic samples; Figure S1: Putative ketamine phase I metabolic products; Figure S2: Putative Cocaine metabolic products; Figure S3: Putative MDMA and MDA metabolic products; Figure S4: Putative Methamphetamine metabolic products; Figure S5: Boxplot of the normalized area of ketamine obtained by LC/MS analysis; Figure S6: Boxplot of the normalized area of ketamine obtained by GC/MS analysis.

## Abbreviations

The following abbreviations are used in this manuscript:

2C-B	4-bromo-2,5-dimethoxyphenetylamine
4-HA	4-Hydroxyamphetamine
4-HMA	4-Hydroxymethamphetamine
ADME	absorption, distribution, metabolism and excretion
ATS	Amphetamine-type substances
BSTFA	N,O-Bis(trimethylsilyl)trifluoroacetamide
CNS	Central nervous system
CPS	classical psychoactive substance
EMIT	Enzyme-multiplied immunoassay technique
ESI	Electrospray ionization
eV	electron volt
GC/MS	Gas chromatography coupled to mass spectrometry
HHA	3,4-Dihydroxyamphetamine
HHMA	3,4-Dihydroxymethamphetamine
HMA	4-Hydroxy-3-methoxyamphetamine
HMMA	4-Hydroxy-3-methoxymethamphetamine
HNK	Hydroxynorketamine
HK	Hydroxyketamine
HRMS	High-resolution mass spectrometry
ISTD	internal standard
LC-MS/MS	Liquid chromatography coupled to tandem mass spectrometry
MDA	3,4-Methylenedioxyamphetamine
MDMA	3,4-Methylenedioxymethamphetamine
MSI	Metabolomics standards initiative
NK	Norketamine
OLS	ordinary least square
ppm	parts per million
THC-COOH	11-nor-9-carboxy-Δ^9^-tetrahydrocannabinol
TMCS	Trimethylchlorosilane
UHPLC	Ultra-high performance liquid chromatography
