# Supplementary material for: Ultra-High-Performance Liquid Chromatography-High Resolution Mass Spectrometry and Gas Chromatography-Mass Spectrometry Screening of Classical Psychoactive Substances, Novel Psychoactive Substances, and Metabolites in Postmortem Urine Samples
Source: Molecules. 2026 Sep 28;31(19):3458. doi: 10.3390/molecules31193458 (PMC13666820; doi:10.3390/molecules31193458)
Supplement: Supplementary file 1 [file molecules-31-03458-s001.zip › molecules-4567752-supplementary.pdf]

Supplementary Materials

Ultra-High-Performance Liquid Chromatography-High Resolution Mass Spectrometry and Gas Chromatography-Mass Spectrometry Screening of classical psychoactive substances, novel psychoactive substances, and metabolites in Postmortem Urine samples

Eleazar Vargas-Mena[1,*], Milton Rosero-Moreano[2], Gonzalo Taborda-Ocampo[2], Julio César España-Amortegui[3], Andrés F. González Suárez[4], Elena E. Stashenko[4]

[1] Grupo de investigación en toxicología forense y ciencias analíticas relacionadas (CROMATOX), Laboratorio de Toxicología Forense, Instituto Nacional de Medicina Legal y Ciencias Forenses, Avenida Las Américas No 98-25, Pereira – Colombia, 660004

[2] Grupo de Inves tigación en Cromatografía y Técnicas Afines (GICTA), Departamento de Química , Facultad de Ciencias Exactas y Naturales, Universidad de Caldas Manizales-Colombia, 170004 ; milton.rosero@ucaldas.edu.co; gonzalo.taborda@ucaldas.edu.co

[3] Research Group for Analytical Food Chemistry, Technical University of Denmark; jceam@food.dtu.dk

[4] Centro de Investigación en Biomoléculas (CIBIMOL), Escuela de Química, Universidad Industrial de Santander, Bucaramanga-Colombia 680001; elenastashenko@gmail.com; andresgonzalez2285@gmail.com

* Correspondence: eleazar.vargas@medicinalegal.gov.co

Table S1. List of potential drugs or metabolites to monitor. Retention times (t_R) and monitored ions (*m/z*) used for the screening gas chromatographymass spectrometry (GC/MS) and ultra-high-performance liquid chromatography-high-resolution mass spectrometry analysis of forensic samples.

Figure S1. Putative ketamine phase I metabolic products.

Figure S2. Putative Cocaine metabolic products.

Figure S3. Putative MDMA and MDA metabolic products.

Figure S4. Putative Methamphetamine metabolic products.

Figure S5. Boxplot of the normalized area of ketamine obtained by LC/MS analysis.

Figure S6. Boxplot of the normalized area of ketamine obtained by GC/MS analysis.

Table S1. List of potential drugs or metabolites to monitor. Retention times (t_R) and monitored ions (*m/z*) used for the screening gas chromatographymass spectrometry (GC/MS) and ultra-high-performance liquid chromatography-high-resolution mass spectrometry analysis of forensic samples.

				**UHPLC-HRMS**		**GC/MS**	
**Name**	**Formula**	**Lote**	**Manufacturer**	**t_R (min)**	**Target *m/z* $[M+H]^+$**	**t_R (min)**	**Target ion *(m/z)***
11-*nor*-9-Carboxy-Δ^9tetrahydrocannabinol	$C_{21}H_{28}O_4$	316.2B9.1	Lipomed	6.33	345.20604	24.8	371<br>473<br>488
4-Bromo-2,5dimethoxyamphetamine	$C_{10}H_{15}NO_2Br$	TRC-B684210	LGC	5.03	260.02817	22.4	44<br>230<br>232
6-Acetylmorphine	$C_{19}H_{21}NO_4$	13-D-04	Sigma	6.62	328.15478	26.9	327<br>286
7-Aminoclonazepam	$C_{15}H_{12}N_3OCl$	106.1B5.1L3	Lipomed	7.12	286.07368	27.1	285<br>256
7-Aminoflunitrazepam	$C_{16}H_{14}N_3OF$	74.1B3.2L2	Lipomed	4.34	284.11542	27.8	283<br>255<br>254
Alprazolam	$C_{17}H_{13}ClN_4$	1096.1B1.1L3	Lipomed	8.67	309.08972	32.03	279<br>308<br>204
Amitriptyline	$C_{20}H_{23}N$	754.1B0.2L1	Lipomed	9.62	278.18974	19.3	58<br>202

Amphetamine	$C_9H_{13}N$	10-D-18	Sigma	3.66	136.11207	11.1	116<br>100<br>44
Benzoylecgonine	$C_{16}H19NO_4$	FE06191501	Cerilliant	4.35	290.13868	24.05	82<br>240<br>105
Bromazepam	$C_{14}H_{10}BrN_3O$	1096.1B1.1L3	Lipomed	6.01	316.00791	25.3	387<br>388
Caffeine	$C_8H_{10}N_4O_2$	131.1B0.1L8	Lipomed	4.00	195.08784	14.9	194<br>109<br>55
Chlorpromazine	$C_{17}H_{19}ClN_2S$	94H1064	Sigma	9.98	319.10311	26.4	58<br>86<br>318
Cocaethylene	$C_{18}H_{23}NO_4$	FE06191501	Cerilliant	5.14	318.16998	24.1	82<br>196<br>105

Table S1. Continued

				UHPLC-HRMS		GC/MS	
**Name**	**Formula**	**Lote**	**Manufacturer**	**t_R (min)**	**Target *m/z* $[M+H]^+$**	**t_R (min)**	**Target ion *(m/z)***
Cocaine	$C_{17}H_{21}NO_4$	FE06191501	Cerilliant	4.65	304.15433	23.4	82<br>182<br>303
Codeine	$C_{21}H_{29}NO_3$	69.1B0.1	Lipomed	6.29	300.15842	25.8	371<br>372<br>178
Desipramine	$C_{21}H_{30}N_2$	SLBG1580V	Sigma Aldrich	9.28	267.1853	23.6	234<br>235<br>116
Diazepam	$C_{16}H_{13}ClN_2O$	1096.1B1.1L3	Lipomed	9.04	285.0783	25.4	256<br>283<br>284

Diltiazem	$C_{22}H_{26}N_2O_4S$	SLBC3562V	Sigma Aldrich	8.96	415.16742	29.4	58<br>71<br>72
Doxepin	$C_{19}H_{21}NO$	096k1405	Sigma	9.07	280.1688	27.1	58<br>165
Ecgonine	$C_9H_{15}NO_3$	206.1B11.2L3	Lipomed	4.5	186.11247	14.5	83<br>82<br>96
Ecgonine ethyl ester	$C_{11}H_{19}NO_3$	FE06191501	Cerilliant	1.55	214.14377	14.2	82<br>83<br>96
Ecgonine methyl ester	$C_{10}H_{17}NO_3$	FE06191501	Cerilliant	1.05	200.12812	13.5	82<br>83<br>96
Fentanyl	$C_{22}H_{28}N_2O$	635.1B12.1	Lipomed	5.48	337.22744	29.2	146<br>245<br>189
Fluoxetine	$C_{17}H_{18}F_3NO$	FN06112123	Supelco	10.02	310.14161	19.08	116
							117
Flurazepam	$C_{21}H_{23}ClFN_3O$	1096.1B1.1L3	Lipomed	8.84	388.05914	25.1	86
Imipramine	$C_{17}H_{24}N_2$	1066.1B0.1L4	Lipomed	9.47	281.2006	19.5	58<br>85<br>234

Table S1. Continued

				UHPLC-HRMS		GC/MS	
**Name**	**Formula**	**Lote**	**Manufacturer**	**t_R (min)**	**Target *m/z* $[M+H]^+$**	**t_R (min)**	**Target ion *(m/z)***
Ketamine	$C_{13}H_{17}NOCl$	663.1B0.3	Lipomed	4.38	238.09946	18.7	180<br>209<br>152

Lidocaine	$C_{14}H_{22}N_2O$	636.1B0.1L6	Lipomed	4.08	235.18063	11.8	86<br>220<br>235
Levomepromazine	$C_{19}H_{24}N_2OS$	R151C0	USP	9.73	329.16864	27.2	58<br>328
MDA	$C_{10}H_{13}NO_2$	79.1B10.2	Lipomed	3.78	180.10191	15.1	116<br>135<br>100
MDMA	$C_{11}H_{15}NO_2$	94.3B10.1	Lipomed	3.82	194.11777	12.07	130<br>135
Meperidine	$C_{15}H_{21}NO_2$	045K1745V	Sigma Aldrich	7.94	248.16314	17.3	247<br>218<br>172
Methamphetamine	$C_{10}H_{15}N$	301.1B6.1	Lipomed	3.74	150.12773	12.05	130<br>220<br>235
Midazolam	$C_{18}H_{13}ClFN_3$	1096.1B1.1L3	Lipomed	5.82	326.08557	27.38	310<br>325<br>312
Morphine	$C_{17}H_{19}NO_3$	35.1B0.1	Lipomed	2.68	286.14377	26.3	429<br>401<br>414
Nordiazepam	$C_{15}H_{11}ClN_2O$	1096.1B1.1L3	Lipomed	8.59	271.0629	23.7	341<br>356<br>313

Table S1. Continued

				UHPLC-HRMS		GC/MS	
**Name**	**Formula**	**Lote**	**Manufacturer**	**t_R (min)**	**Target *m/z* $[M+H]^+$**	**t_R (min)**	**Target ion *(m/z)***

Norfentanyl	C14H21N2O	635.1B12.1	Lipomed	4.42	233.16484	28.7	155<br>154
Norketamine	$C_{12}H_{15}NOCl$	932.1B3.1	Lipomed	4.35	224.08367	19.4	238<br>210<br>225
Oxazepam	$C_{15}H_{11}N_2O_2$	1096.1B1.1L3	Lipomed	6.58	287.05818	24.8	429<br>430<br>431
PCP	$C_{17}H_{25}N$	436.1B3.1	Lipomed	6.92	244.20609	20.1	200<br>242
Sertraline	$C_{17}H_{17}Cl_2N$	1068.1B0.1L5	Lipomed	9.89	306.0805	25.7	274<br>276
Temazepam	$C_{16}H_{13}ClN_2O_2$	1096.1B1.1L3	Lipomed	8.71	301.07314	23.7	343<br>257<br>283
α-hydroxyalprazolam	$C_{17}H_{13}ClN_4O$	1096.1B1.1L3	Lipomed	8.15	325.08378	32.1	381<br>383<br>396
α-hydroxymidazolam	$C_{18}H_{13}ClFN_3O$	1096.1B1.1L3	Lipomed	6.2	342.01773	30.1	310<br>398<br>413

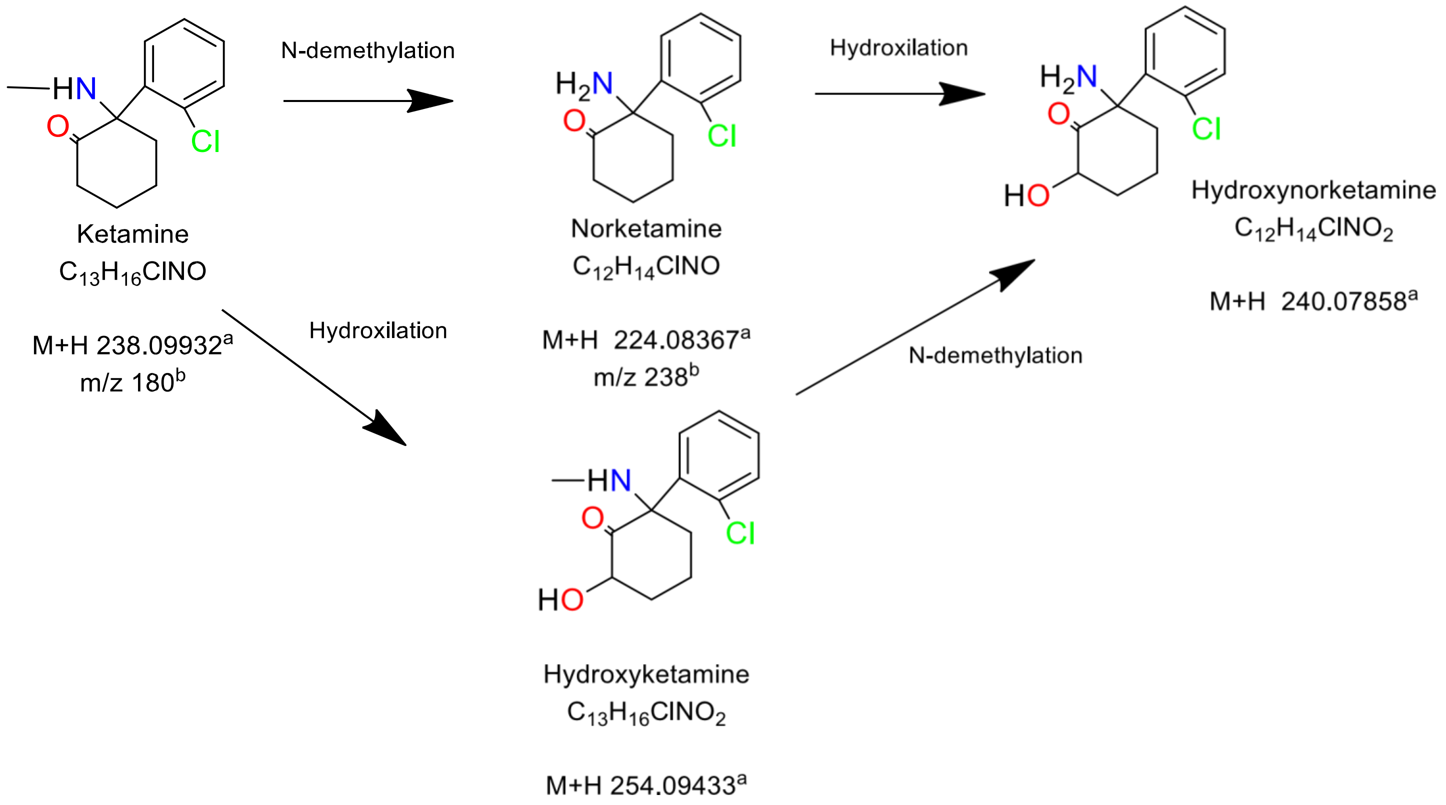

Figure S1. The main metabolites of ketamine found in postmortem urine. [a] corresponds to the protonated molecule $[M+H]^+$ identified by LC/MS analysis, and [b] corresponds to the base peak in the GC/MS spectra. Hydroxyketamine and hydroxynorketamine were not identified in GC/MS analysis.

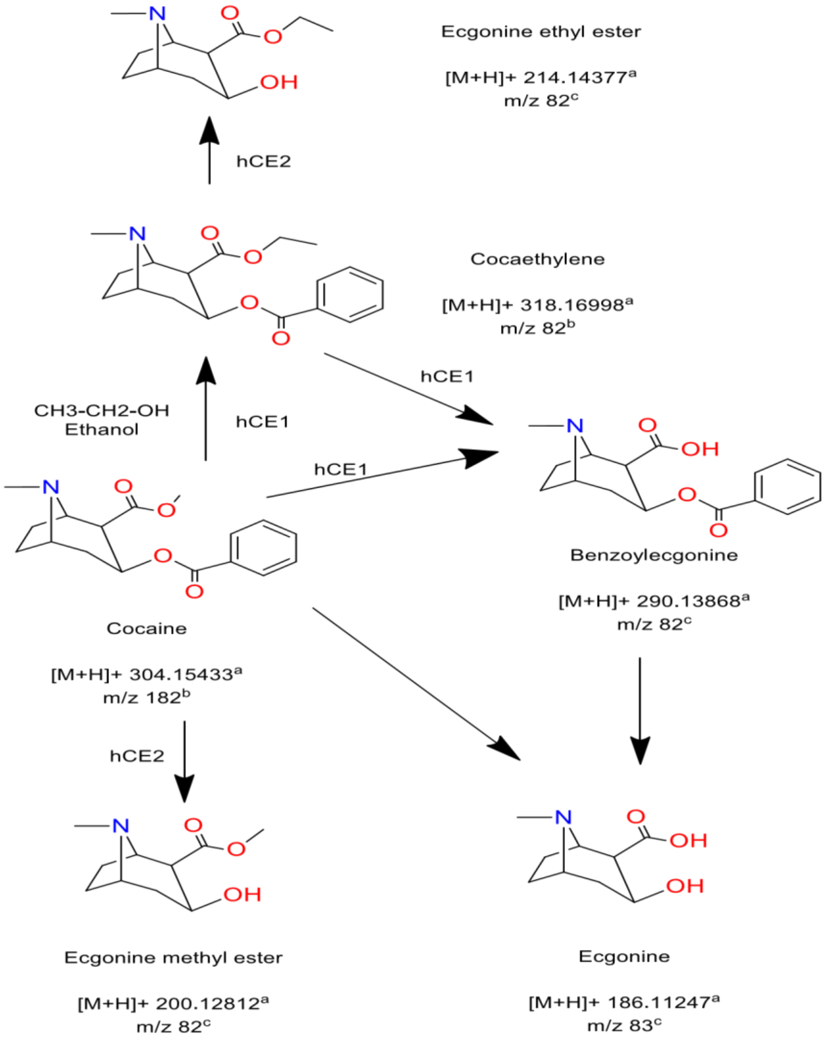

Ecgonine ethyl ester
[M+H]+ 214.14377a
m/z 82c
hCE2
Cocaethylene
[M+H]+ 318.16998a
m/z 82b
hCE1
CH3-CH2-OH
Ethanol
hCE1
hCE1
Benzoylecgonine
[M+H]+ 290.13868a
m/z 82c
Cocaine
[M+H]+ 304.15433a
m/z 182b
hCE2
Ecgonine methyl ester
[M+H]+ 200.12812a
m/z 82c
Ecgonine
[M+H]+ 186.11247a
m/z 83c

Figure S2.

The main metabolites of cocaine found in postmortem urine. [a] corresponds to the protonated molecule $[M+H]^+$ identified by LC/MS analysis, [b] corresponds to the base peak in the GC/MS spectra, and [c] corresponds to the base peak of the silylated adduct in the GC/MS spectra. hCE1 and hCE2 are Human Carboxylesterase 1 and 2, respectively.

N-demethylation

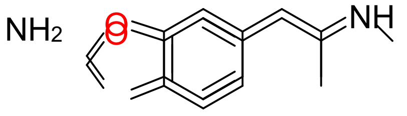

O

3,4-methylenedioxymethamphetamine ***MDMA***

M+H 194.11756[a] m/z
180[b]

3,4-methylenedioxyamphetamine ***MDA***

M+H 180.10191[a] m/z
44[b]

O

O-demethylation
O-demethylation

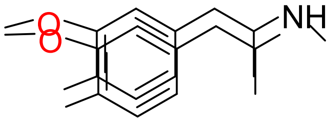

HO

4-hydroxy-3-methoxymethamphetamine ***HMMA***

M+H 196.13312[a]

4-hydroxy-3-methoxyamphetamine ***HMA***

M+H 182.11772[a]

NH2

HO

Figure S3.

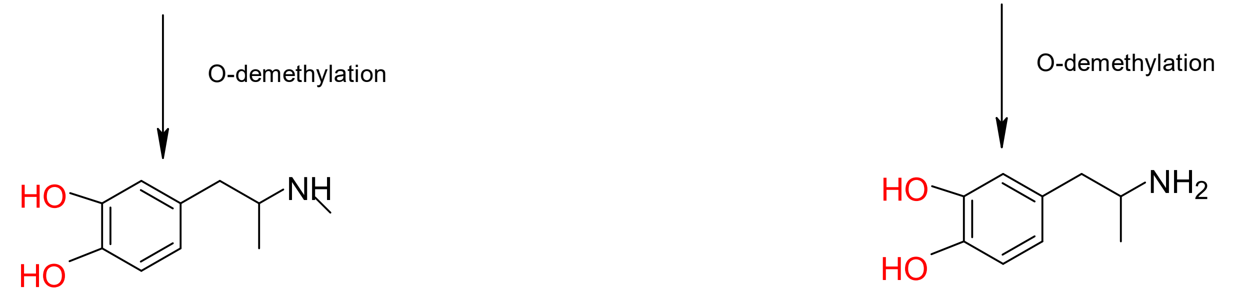

3,4-dihydroximethamphetamine ***HHMA***

3,4-dihidroxyanfetamina ***HHA***

M+H 182.11756[a]

M+H 168.10191[a]

The main metabolites of MDMA and MDA found in postmortem urine. [a] corresponds to the protonated molecule $[M+H]^+$ identified by LC/MS analysis, and [b] corresponds to the base peak in the GC/MS spectra.

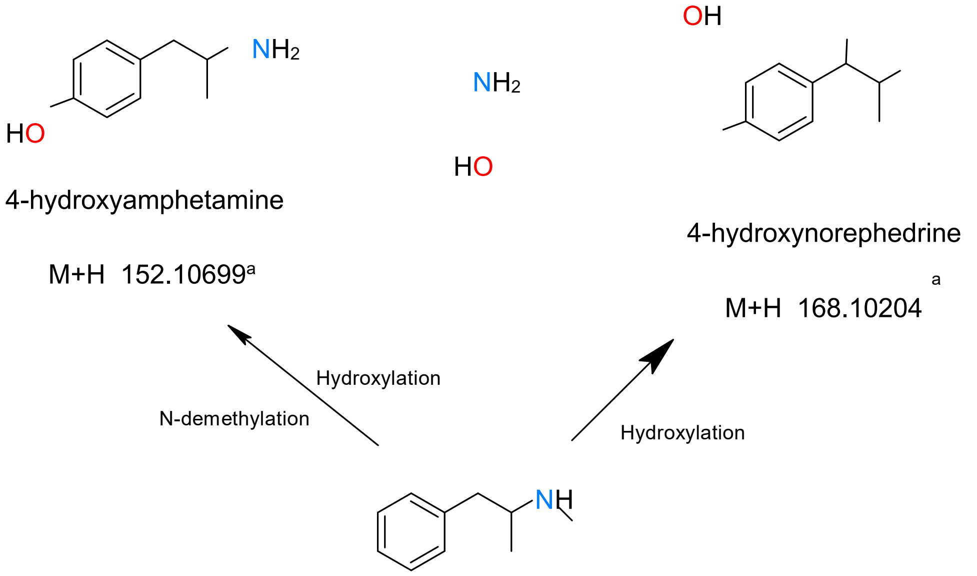

Methamphetamine ***MA***

Figure S4.

M+H 150.12773[a]

[b] m/z 130

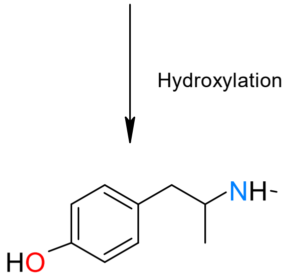

4-hydroxymethamphetamine

M+H 166.12280[a]

The main metabolites of Methamphetamine found in postmortem urine. [a] corresponds to the protonated molecule $[M+H]^+$ identified by LC/MS analysis, and [b] corresponds to the base peak in the GC/MS spectra.

Figure S5.

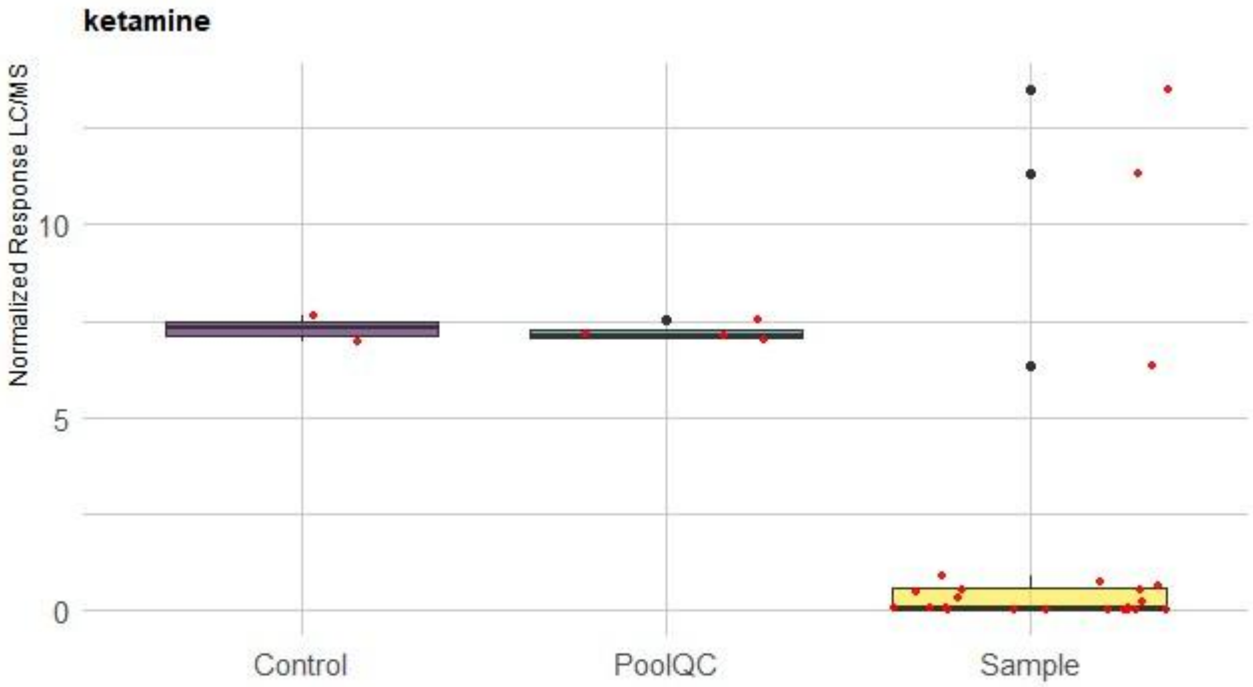

Figure S5. Boxplot of the normalized area of ketamine obtained by LC/MS analysis. RSD in controls (n=2) and poolQCs (n=4) is lower than 15 %. Variability in samples is expected to be higher than in controls and pool QCs. Normalized response (area) of ion 180 m/z of ketamine

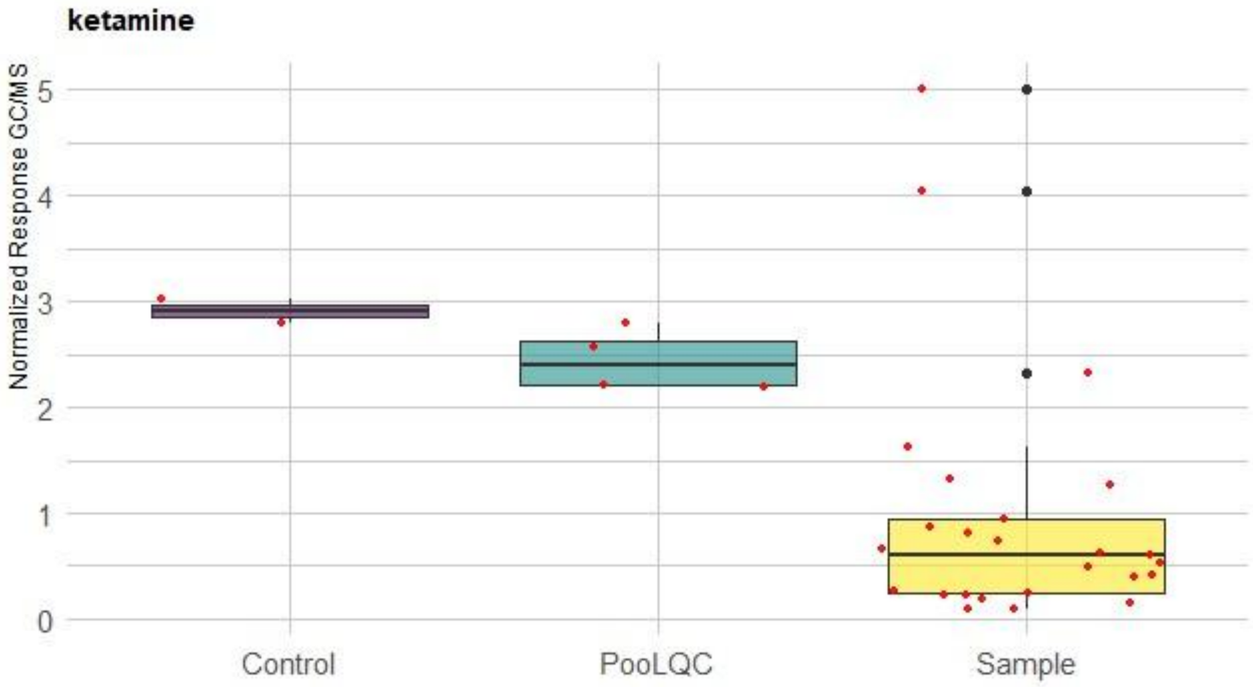

Figure S6. Boxplot of the normalized area of ketamine obtained by GC/MS analysis. RSD in controls (n=2) and poolQCs (n=4) is lower than 15 %. Variability in samples is expected to be higher than in controls and pool QCs. Normalized response (area) of ion 180 m/z of ketamine
